# Prediction of Drug Positioning for Quan-Du-Zhong Capsules Against Hypertensive Nephropathy Based on the Robustness of Disease Network

**DOI:** 10.3389/fphar.2019.00049

**Published:** 2019-02-12

**Authors:** Feifei Guo, Wen Zhang, Jin Su, Haiyu Xu, Hongjun Yang

**Affiliations:** ^1^Institute of Chinese Materia Medica, China Academy of Chinese Medical Sciences, Beijing, China; ^2^College of Ethnic Medicine, Chengdu University of Traditional Chinese Medicine, Chengdu, China

**Keywords:** hypertensive nephropathy, *Eucommia ulmoides*, drug positioning, network robustness, network pharmacology

## Abstract

Hypertensive nephropathy (HN) is a medical condition in which chronic high blood pressure causes different kidney damage, including vascular, glomerular and tubulointerstitial lesions. For HN patients, glomerular and tubulointerstitial lesions occur in different renal structure with distinct mechanisms in the progression of renal damage. As an extraction of *Eucommia ulmoides*, Quan-du-zhong capsule (QDZJN) has the potential to treat HN due to antihypertensive and renal protective activities. Complicated mechanism of HN underlying various renal lesions and the “multi-component and multi-target” characteristics of QDZJN make identifying drug positioning for various renal lesions of HN complex. Here, we proposed an approach based on drug perturbation of disease network robustness, that is used to assess QDZJN positioning for various HN lesions. Topological characteristics of drug-attacked nodes in disease network were used to evaluated nodes importance to network. To evaluate drug attack on the whole disease network of various HN lesions, the robustness of disease networks before/after drug attack were assessed and compared with null models generated from random networks. We found that potential targets of QDZJN were specifically expressed in the kidneys and tended to participate in the “inflammatory response,” “regulation of blood pressure,” and “response to LPS and hypoxia,” and they were also key factors of HN. Based on network robustness assessment, QDZJN may specifically target glomeruli account to the stronger influence on glomerular network after removal of its potential targets. This prediction strategy of drug positioning is suitable for multi-component drugs based on drug perturbation of disease network robustness for two renal compartments, glomeruli and tubules. A stronger influence on the disease network of glomeruli than of tubules indicated that QDZJN may specifically target glomerular lesion of HN patients and will provide more evidence for precise clinical application of QDZJN against HN. Drug positioning approach we proposed also provides a new strategy for predicting precise clinical use of multi-target drugs.

## Introduction

Hypertension is a disease that leads to organ damage. Renal damage is a common lesion induced by hypertension due to the interaction of multiple factors, including blood pressure, endothelial dysfunction, the RAAS, ROS, and inflammation (Imig et al., 2018). Hypertensive nephropathy (HN) is a medical condition in which chronic high blood pressure causes kidney damage, including damage to two renal compartments, the glomerular and tubulointerstitial compartments. Exploration of interactions between glomerular and tubulointerstitial compartments contributing to HN creates opportunities to find better therapeutic strategies for renal protection against organ damage in hypertension. For HN patients, glomerulosclerosis and tubulointerstitial fibrosis occur in different renal structure with distinct mechanisms in the progression of renal damage. At the level of the tubulointerstitial compartment, it constitutes 95% of the total kidney mass (Berthier et al., 2012). Early tubular injury is caused by renovascular hypertension, leading to tubular cell proliferation and deposition of matrix proteins primarily within the interstitium which extends far beyond the glomeruli (Mai et al., 1993). In some hypertension patients, circulating antibodies and immunoglobin G deposit along the tubular basement membranes (Mai et al., 1993). Thus, tubulointerstitial change is regarded as a determinative factor in the development of renal damage (Nath, 1992) and may be the important initial site of injury (Mai et al., 1993). At the level of the glomeruli, increased blood pressure leads to increased capillary pressure which results in capillary stretching, endothelial damage, and breakdown of the capillary barrier (Folkow et al., 1977’ Bidani and Griffin, 2004). This leads to increased glomerular protein filtration that causes segmental necrosis and glomerulosclerosis (Shankland, 2006). Glomerular sclerosis and preglomerular vascular structural alterations can cause a further reduction in renal blood flow and enhance the progression of hypertensive renal injury (Folkow et al., 1977; Campese et al., 1991; Shankland, 2006; Hemmelgarn et al., 2010).

Numerous drugs are used to control blood pressure, including β-blockers, vasodilators, renin-angiotensin system inhibitors and diuretics. The clinical strategy for treatment of HN is to achieve and maintain blood pressure using minimal drug combinations with minimal side effects (Halbach, 2018). However, in later periods, hypertension patients become less responsive to drugs, and drug combinations are associated with significant side effects (Yusuf et al., 2008; Parving et al., 2012; Fried et al., 2013). It is a challenge to find the best treatment for HN patients that can balance the positive and negative effects of drugs. In China, EU has been widely used to treat hypertension and has also been used in Chinese traditional medicine as a folk drink and functional food for several thousand years (Hussain et al., 2016). EU is a plant containing various chemical constituents such as lignans, iridoids, phenolics, steroids, flavonoids, and other compounds; these components of EU possess various medicinal properties, such as antioxidant, anti-inflammatory, and anti-allergic properties, as well as blood pressure control (Kulomaa et al., 1997; Yen and Hsieh, 1998; Hsieh and Yen, 2000; Park et al., 2006; Bonghyun et al., 2009). *Eucommia* bark extract is a vasorelaxant used for antihypertensive formulations (Kwan et al., 2003; Luo et al., 2010; Greenway et al., 2011). Extract of EU has also been reported to reduce the concentration of hydrogen peroxide in the kidneys and to protect against renal injury (Park et al., 2006; Liu et al., 2012). Thus, EU has the potential to treat HN because of its antihypertensive and renal protective activities with fewer side effects.

However, multicomponents and various medicinal properties make it complex to illustrate the effect of EU on renal protection and blood pressure control. More importantly, the architecture of the kidney increases the complexity. It is difficult to elucidate the mechanisms of EU in treating HN because this involves connecting a multi-component drug with various properties to a complex disease with various risk factors. Based on the “multi-component and multi-target” principle, network robustness methods can be learned from network sciences to identify drug positioning for various HN lesions. Complex systems of disease can be described by networks, in which gene interactions in specific disease conditions are represented by vertices and edges between vertices. Drug disturbance can be presented as an attack on the disease network. Drug effects on system robustness can be addressed by analyzing how the network architecture changes as drug-attacked vertices are removed. In the field of network science, network robustness against perturbations provides a standard of measurement for assessing multi-drug attacks on disease network. Health systems are generally robust against various perturbations but can be fragile when faced with perturbations for which the system has not been optimized (e.g., disease conditions) (Kitano, 2007). Drug attacks with stronger effects on the reduction of the robustness of the disease network suggest that this drug maybe more effective for this disease or pathological process.

Based on the various lesions of HN and the genes expressed in different compartments of kidneys of HN patients, we proposed an approach based on the drug perturbation of disease network robustness of two renal compartments, which is suitable for the assessment of multicomponent drug positioning. The drug perturbation of two different disease networks, renal glomeruli and tubules, will be helpful in describing clinical features and applications of drugs. As an example of a multicomponent drug, an extraction of EU called the QDZJN, which is approved by CFDA, was used in our study. We found that potential targets of QDZJN were specifically expressed in the kidneys and tended to participate in the inflammatory response, regulation of blood pressure, and response to LPS and hypoxia, which were also key factors of HN. Based on network robustness assessment, QDZJN may specifically target glomeruli, suggested by its stronger influence on the glomerular network after removal of its potential targets. QDZJN may be used for HN patients with glomerular injury for better pharmaceutical effects. These finding will provide more evidence for the precise clinical application of QDZJN against HN. This approach we proposed maybe helpful for the precision clinical use of multi-target drugs against HN and other complex diseases.

## Materials and Methods

### Literature Mining of Component Compounds of QDZJN

QDZJN is a CFDA-approved drug that is the extraction of EU. The 84 identified component compounds of QDZJN were retrieved by literature mining of research papers about the bioactive constituents of EU. “*Eucommia ulmoides*,” “du zhong,” “compounds,” “chemical” and related synonyms were used to retrieve relevant literatures. Information on the component compounds collected from the literature was tidied and reorganized. The molecular structure files of all the component compounds of QDZJN were downloaded from the ChemSpider database^1^ and saved in InChI format. Detailed information on the constituent components of QDZJN is provided in Supplementary Table S1.

### Target Prediction of QDZJN’s Component Compounds

Target prediction of the component compounds was executed by BATMAN-TCM (Liu et al., 2016), which is a web service for discovering the therapeutic mechanisms of multicomponent drug (Liu et al., 2016). In BATMAN-TCM, potential drug-target interactions were sorted based on possibility scores from largest to smallest. Possibilities of drug-target interactions were predicted according to their similarities to known drug-target interactions that were previously published and validated (Liu et al., 2016). Three types of validation methods, including “leave-one-interaction-out” cross-validation, “leave-one-drug-out” cross-validation and validation of the independent test set, were applied to measure the performances. Th results showed that BATMAN-TCM had satisfactory performance for target prediction. In this study, potential targets of 84 compounds for QDZJN were predicted with possibility scores larger than 20.

### Annotation Enrichment Analysis of Gene Tissue Specificity and Biological Function

Tissue specificity of potential targets of QDZJN was analyzed by the DAVID functional annotation of tissue expression (“Uniprot Tissue”) and GO functional enrichment analysis of DAVID was used to detect enriched biological processes of genes (Dennis et al., 2003), both based on the hypergeometric cumulative distribution test. Annotation terms with *p*-values < 0.05 and fold enrichment larger than 1 were considered statistically significant.

### Disease Genes of Hypertension and HN

1409 disease genes related to hypertension were downloaded from DisGeNET Database via MeSH term D006973 (Piñero et al., 2017). And 57 disease genes related to HN were downloaded from DisGeNET via MeSH terms D006977 and D006978 (downloaded on December 3rd, 2018).

### Differentially Expressed Genes From Renal Compartments of HN Patients

Transcriptome data were collected from tubulointerstitial and glomerular compartments from kidney biopsies of HN patients (*n* = 15) and healthy living donors (*n* = 27) in Berthier’s study (Berthier et al., 2012). In this study, 12,025 human genes were expressed above the 27 Poly-A Affymetrix control expression baseline (negative controls) in the glomerular and tubulointerstitial compartments and were used for further analyses. Microarray data from HN patients will be available on the GEO web site under accession numbers GSE37455 (tubulointerstitial) and GSE37460 (glomeruli). For the microarray data, unpaired statistical analyses for each comparison between the relevant study groups were performed using the significance analysis of microarrays method. DEGs of glomeruli and tubules compared to those of healthy samples were defined by a *p*-value < 0.05 and with a fold change ≥ 1.1 for the upregulated genes and ≤0.91 for the downregulated genes, which were considered significant and used for further transcriptional and pathway analyses.

### Construction of Disease Networks for Two Renal Compartments

Disease networks consisted of DEGs and interactions between DEGs. Protein interactions from STRING (Szklarczyk et al., 2017) (version 10) whose confidence scores were larger than 0.4 were used to separately construct protein–protein interaction networks of DEGs of glomerular and tubulointerstitial compartments of HN patients (downloaded on March 19, 2018). Cytoscape (version 3.4.0) was utilized to visualize the networks and calculate the topological characteristics of nodes, including degree, APL, and CC_node_ (Su et al., 2014). Node size is correlated with node degree in networks. Nodes with degrees twofold larger than the median degree of all nodes were defined as hub nodes. Some nodes were also potential targets of QDZJN, these were marked by red edges and ere regarded as nodes attacked by the drug.

### Network Robustness Represented by Topological Characteristics of Nodes

First, two characteristics of nodes were used to evaluate the importance of drug-attacked nodes in networks, including APL and CC_node_ of node. Average length of shortest path (APL_node_) is a characteristic of network nodes that provides a more sophisticated view than the node degree of local connections by also considering the degree of a node’s neighbors. This approach accounts for the fact that all edges are not equal: connections to a highly connected node may render a node more significant or influential (Baldassano and Bassett, 2016). The shorter the path length is, the more important the node is.

The average shortest path length of source node is

$${APL}_{node\left(s\right)=\underset{t\in V}{\Sigma }\frac{d\left(s,t\right)}{n},}$$

where *V* is the set of nodes in *G*, *d*(*s*, *t*) is the shortest path from *s* to *t*, and *s* is the source node. *n* is the number of nodes in *G.*

Closeness centrality of a node *s* is the reciprocal of the sum of the shortest path distances from *v* to all *n*-1 other connected nodes. Since the sum of distances depends on the number of nodes connected with source node *s*, closeness is normalized by the sum of minimum possible distances *n*-1,

$${CC}_{node\left(s\right)=\frac{n-1}{\Sigma_{v=1}^{n-1}d\left(s,v\right)},}$$

where *d*(*s*, *v*) is the shortest-path distance between *s* and *v*, and *n* is the number of nodes connected with *s* in the graph. The larger the CC_node_ is, the more important the node is.

Here, a comparison of drug-attacked nodes and other nodes in disease network provides a more comparative view of drug-attacked node topological networks, and a unpaired *t*-test was used to assess differences between two kinds of nodes; a *p*-value < 0.05 was considered significant.

### Network Robustness Represented by Topological Characteristics of Networks

Drug attack on networks were evaluated by changes of network’s topological characteristics after removal of the drug target. The Average path length (APL_net_) is one of the most robust measures of network topology, along with its CC_net_.

The average shortest path length of a whole network is

$${APL}_{net=\underset{s,t\in V}{\Sigma }\frac{d\left(s,t\right)}{n\left(n-1\right)},}$$

where *V* is the set of nodes in *G*, *d(s, t)* is the shortest path from *s* to *t*, and *n* is the number of nodes in *G*.

The clustering of a node *v* is the fraction of possible triangles through that node that exist,

$$C_{v=\frac{2T\left(v\right)}{\deg(v)\left(\deg(v)-1\right)},}$$

where *T(v)* is the number of triangles through node *v* and *deg(v)* is the degree of *v*. The CC_net_ for the graph *G* is the average,

$${CC}_{net=\frac{1}{n}\underset{v\in G}{\Sigma }C_{v},}$$

where *n* is the number of nodes in *G*.

In our study, these two indicators were used to evaluated network robustness, including the APL and the CC_net_ of the whole network. First, the average path length distinguishes a stable network from one, with a shorter average path length being more robust. Therefore, the quotient of the APL before/after attack was used to evaluate the influence of drug attack on the disease network. The larger the APL was, the less stable the network and, the larger the influence made by the drug. Similarly, the quotient of the CC_net_ before/after attack was also used to evaluate network robustness after a drug attack. A smaller network CC_net_ indicated a less stable network and a larger drug influence.

### Null Model of Random Network and Permutation Test of Real and Random Networks

To assess drug attack effects on network robustness, the network robustness indicators (RI) of random networks were generated as a null model. Drug attack to a real disease network was compared with null models with these two indicators (APL_net_ and CC_net_) to investigate whether there were significant differences of network robustness between real network and random network after same drug attack. Null models were generated for the disease networks through randomization of network with preserved node and edge numbers of real network. 1,000 random networks were generated as a benchmark, and 1,000 indicators were generated after the same drug attack on random networks as a null distribution for the permutation test (Baldassano and Bassett, 2016). The indicator of a real network out of a 95% confidence interval of null distribution was regarded as having a significant difference (*p*-value < 0.05 of permutation test). Comparing to the null distribution generated from 1,000 random networks, drug attack that make a significant disturbance in the real disease network was significative. The *p*-value of drug attack on a real network can be used as a network robustness index for comparison. Smaller *p*-values indicate larger network disturbance drug attacks.

### Construction of Hierarchical Network for QDZJN Compounds, DEGs of Glomeruli and HN Related Process

A hierarchical network of chemical compounds, potential targets and pathological processes was constructed, including component compounds and potential targets of QDZJN, HN related biological processes, drug-target interactions predicted by BATMAN-TCM, relationships between targets and pathological processes of HN retrieved from literatures. Potential targets of QDZJN were also DEGs of glomeruli, namely, drug-attacked nodes in the glomerular network.

## Results

### QDZJN’s Potential Targets Were Upregulated in the Kidney and May Have Anti-hypertension Activity Based on Functional Enrichment Analysis

QDZJN is a CFDA approved drug for hypertension. As multicomponent drug extracted from EU, 84 component compounds of QDZJN were collected via literature mining, including 26 lignans, 17 iridoids, 22 phenylpropanoids, 13 flavonoid, and 8 other kinds of compounds. The structures and other detailed information of the component compounds of QDZJN are shown in the Supplementary Table S1. The 427 potential targets of 84 compounds were predicted by BATMAN-TCM (Liu et al., 2016).

**FIGURE 1:**
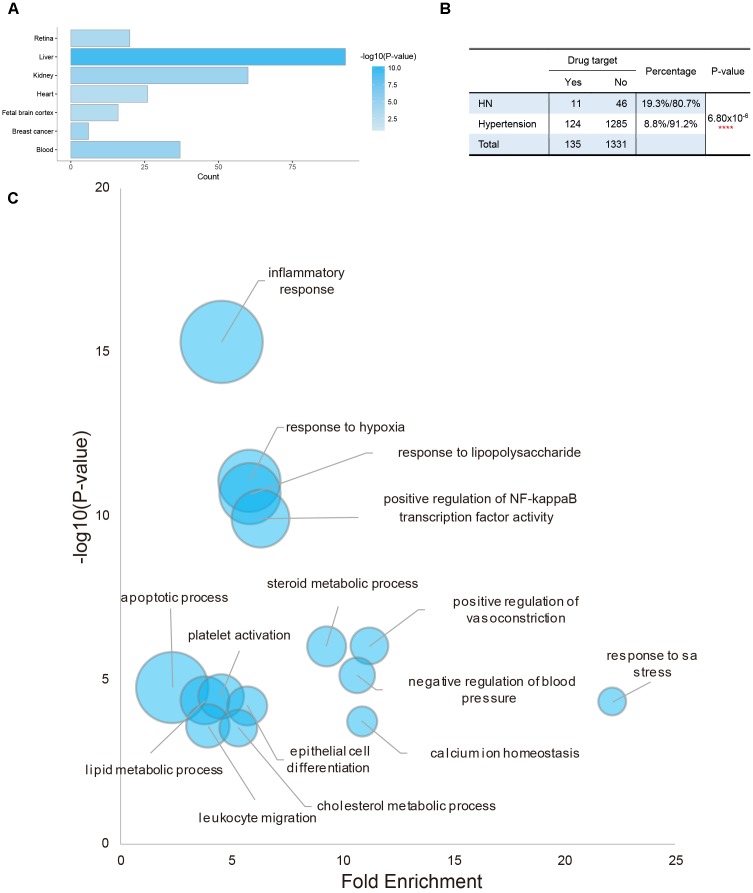
Tissue enrichment and functional enrichment of potential targets of QDZJN. **(A)** Histogram of possible target tissue of QDZJN’s potential targets by tissue expression enrichment in DAVID. *X*-axis is the count of targets, and color gradient of bar is related with *p*-value of enrichment analysis. **(B)** Chi-square test table for comparison of the distribution of QDZJN’s targets in disease gene of hypertension and HN. **(C)** Bubble diagram of enriched GO terms of QDZJN’s targets. *X*-axis is fold enrichment, *Y*-axis is related with *p*-value of enrichment analysis. Bubble size changes with the count of targets in this term.

Tissue specific expression of potential targets was investigated to explore target tissue attacked by QDZJN. Enriched tissue terms within the UP_Tissue (“Uniprot Tissue”) were detected using DAVID functional annotation of tissue expression (Dennis et al., 2003). Interestingly, potential targets of QDZJN were enriched in UP_TISSUE terms including liver and kidney, among which 60 potential targets were expressed in a high level in normal kidney tissue (Figure 1A). Besides, 1409 disease genes related to hypertension and 57 disease genes related to HN were downloaded from DisGeNet Database. A chi-square test was used to compare the distribution of drug target in disease gene of hypertension and HN. A small *P*-value indicates that potential drug targets of QDZJN are enriched in disease genes in HN than hypertension (*p*-value = 6.80 × 10^-6^, Figure 1B). Even though more drug targets are also hypertensive genes, there are 19.3% disease genes of HN targeted by QDZJN which significantly larger than this proportion in hypertension. This result suggests that kidney may be one of the target organs of QDZJN and HN may be a proper indication of QDZJN than hypertension which was in agreement with the renal protection activity of EU. Functional enrichment analysis of GO terms showed that QDZJN potential targets were enriched in biological processes including “inflammatory response,” “response to hypoxia,” “response to LPS,” “regulation of vasoconstriction and blood pressure” (Figure 1C). Consistent with this finding, EU has been reported to exhibit anti-oxidant, anti-hypertensive, and anti-inflammatory activity.^18,30,^ ^31^ According to the above result, QDZJN has the potential to prevent kidney damage induced by hypertension, as well as hypertension. Of note, a randomized controlled study of QDZJN showed that this drug was effective for the treatment of renal hypertension (Zhen and Liang, 2012; Jing et al., 2015).

### Differentially Expressed Genes in Glomeruli and Tubules of HN Patients

**FIGURE 2:**
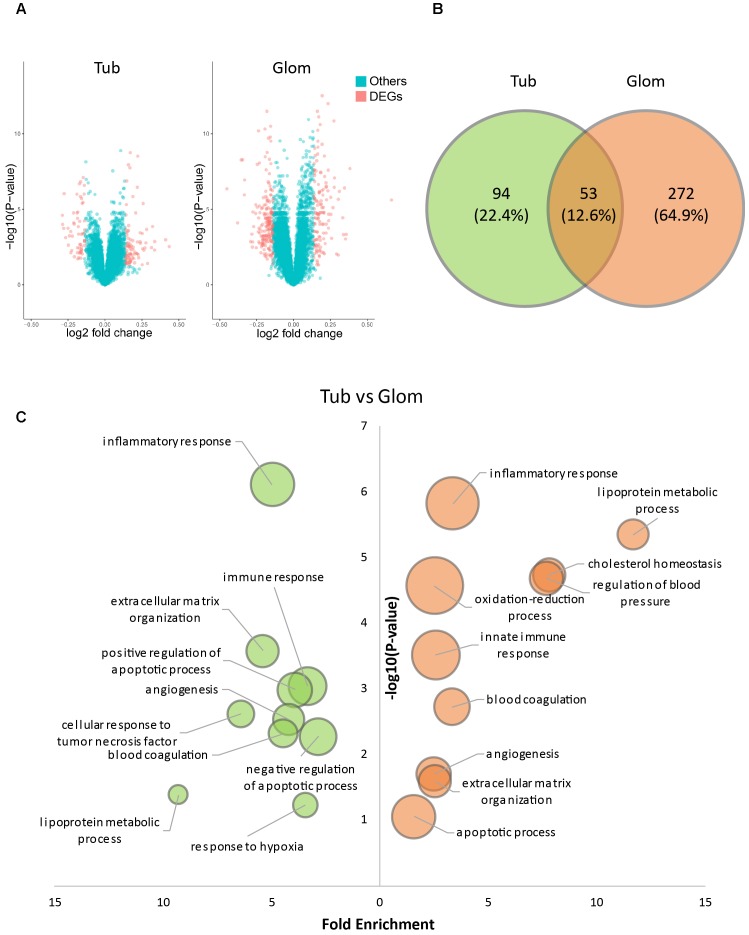
Differentially expressed genes (DEGs) in tubules and glomeruli of HN patients. **(A)** Volcano plot of differentially expressed genes in tubules and glomeruli. **(B)** Venn diagram of DEGs in tubules (green) and glomeruli (orange). **(C)** Bubble plot of enriched GO terms of DEGs of tubules (green) and glomeruli (orange). *X*-axis is fold enrichment, *Y*-axis is related with *p*-value of enrichment analysis. Bubble size changes with the count of targets in this term.

Glomerular and tubulointerstitial lesion are two common types of kidney damage induced by hypertension. To investigate the relationship between QDZJN and two kidney compartments, gene expression data were collected from kidney tubules and glomerular samples of HN patients and healthy living donors in Berthier’s study (Berthier et al., 2012). DEGs were identified from comparing the kidneys of human with HN with healthy kidneys, as the kidney maybe QDZJN’s target organ. Kidneys from HN patients demonstrated 325 glomerular and 147 tubulointerstitial genes with significantly changed mRNA expression compared with healthy kidneys (Figure 2A). A total of 131 upregulated and 194 downregulated genes were identified in glomeruli, and 90 upregulated and 57 downregulated genes were identified in tubule (Figure 2A). There were 53 genes differentially expressed in both glomerular and tubulointerstitial compartments (Figure 2B). Result of enrichment analysis showed that glomeruli and tubules shared some pathways, e.g., “inflammatory and immune response,” “apoptotic process,” “angiogenesis,” and “response to oxidative stress” (Figure 2C) which are key factors in renal damage induced by hypertension (Imig et al., 2018). DEGs in glomeruli specifically participate in the regulation of blood pressure and cholesterol homeostasis which is related to hypothalamic-pituitary-adrenal (HPA) axis. Similar to DEGs, biological processes related to “inflammatory response,”“response to hypoxia,” and “regulation of blood pressure” were also enriched in QDZJN’s potential targets. This finding implied that QDZJN intervenes in renal damage by controlling responses to inflammation, oxidative stress and blood pressure.

### Construction of Disease Networks of Kidney Glomeruli and Tubules

**FIGURE 3:**
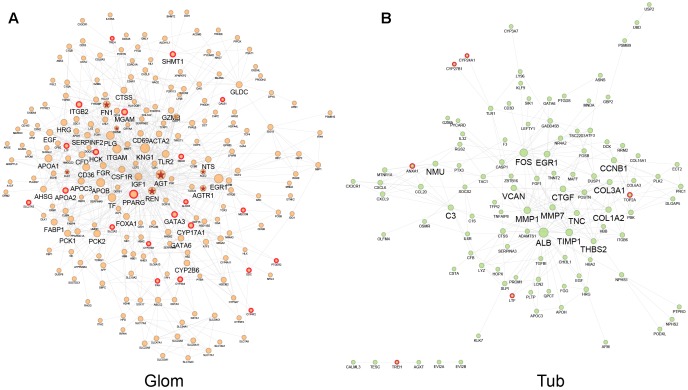
Interaction network of DEGs in glomeruli **(A)** and tubules **(B)**. Node size changes with the node degree in network. The hub nodes were marked with big name labels and potential drug targets of QDZJN were marked with red edges. Nodes with red star label were disease genes related to HN from DisGeNET database.

Disease networks based on DEGs in glomeruli and tubules were constructed to exhibit the interaction between DEGs in two kidney compartments and to assess the drug perturbation of disease networks. The glomerular disease network consisted of DEGs in the glomerulus and interactions between DEGs from STRING database, containing 234 nodes with 836 edges (Figure 3A). The tubule disease network contained 109 nodes with 280 edges (Figure 3B). Node size is relevant to the degree of node in the disease network, which is defined as the number of edges connected to that node (see “Materials and Methods” section). Hubs are important in the disease network. After the hub is attacked by drug, it broadcasts the effect to the nodes to which it is linked. Nodes with degree two-fold larger than median of degrees of all nodes were defined as hub nodes which play important roles. There were 15 and 44 hub nodes in tubular and glomerular networks, respectively. ALB, FOS and EGR1 were both hub nodes in two networks, these genes play critical roles in the progression of HN. Based on previous studies, urinary albumin (ALB) had the potential to be a marker for hypertension (Takase et al., 2015). EGR1 deficiency protects against renal function by attenuating NF-κB and TGFβ-mediated renal inflammation/fibrosis (Ho et al., 2016, 1). However, most hub nodes were specifically expressed in single renal compartment.

To investigate the importance of drug-attacked nodes in the disease network, potential targets were marked with red edges in Figure 3. In the glomerular and tubulointerstitial network, 6 and 22 nodes, respectively, were potential targets of QDZJN and were regarded as drug-attacked nodes that were attacked by QDZJN’s compounds (Figure 3). In the glomerular network, hub node PPARG was targeted by QDZJN, it has been reported to be a new target for the treatment of hypertension as well as pivotal in vascular muscle as a regulator of vascular structure, vascular function, and blood pressure (Leibovitz and Schiffrin, 2007). In both tubulointerstitial and glomerular networks, LTF was a key node that has been verified to be an antihypertensive peptide (Ruiz-Giménez et al., 2012). QDZJN attacked 8 hub nodes in the glomerular network, but 0 node in the tubular network. This result suggested that potential targets of QDZJN may play more important roles in glomerular network.

### Drug Attack of QDZJN on Disease Networks Based on Topological Characteristics of Nodes

**FIGURE 4:**
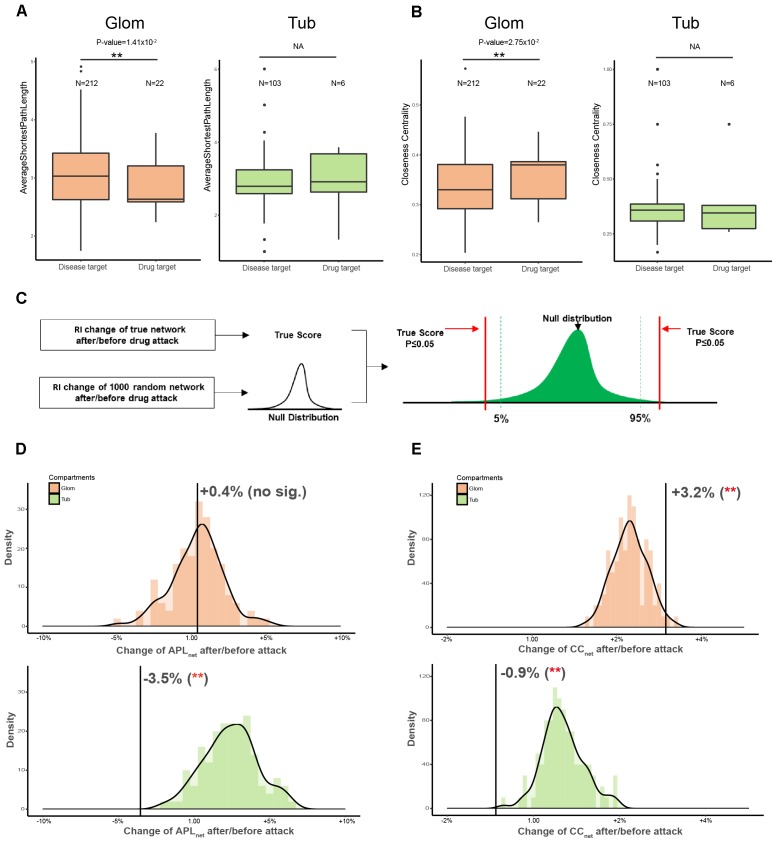
Comparison of topological features of drug-attacked node in tubulointerstitial and glomerular network. **(A,B)** Boxplot of APL_node_ **(A)** and CC_node_ **(B)** of drug-attacked nodes and other nodes in tubulointerstitial and glomerular network. ^∗∗^*p*-value < 0.05. **(C)** Schematic diagram of Permutation test. Network topological features were used as robustness indicators (RI) of networks. Null distribution of RI of 1000 random network was generated to maintain the same edge number of the true network and randomize interactions between nodes. Comparing with the null distribution, statistical significance of RI of true network can be calculated. **(D,E)** Robustness (APL_net_ for **D**, CC_net_ for **E**) of glomerular and tubulointerstitial network versus random network (green distribution for tubules and orange distribution for glomeruli). *X*-axis is the percentage change of robustness indicators before/after attack. ^∗∗^*p*-value < 0.05.

QDZJN affected eight hub nodes of the glomerular network which indicated that drug-attacked nodes may play important roles in the glomerular network. To comprehensively evaluate the importance of drug-attacked nodes in glomerular and tubulointerstitial disease network, two more topological features of drug-attacked nodes were compared with other nodes in the disease network, including average path length and CC_node_ of node. The average path length of node (APL_node_) is defined as the average number of steps along the shortest paths for all nodes connected to that node. It is a measure of the efficiency of information or mass transport on a network. CC_node_ of a node is a measure of centrality in a network, calculated as the sum of the length of the shortest paths between the node and all other nodes in the graph. The more central a node is, the closer it is to all other nodes. In glomerular network, drug-attacked nodes have relative shorter APL_node_ and larger CC_node_ than other nodes in the glomerular network with statistically significant difference (*p*_apl_ = 1.42 × 10^-2^; *p*_cc_ = 2.75 × 10^-2^) indicating that drug-attacked nodes tend to connect closely to nodes in the network (Figure 4A,B). However, APL_node_ and CC_node_ of drug-attacked nodes in tubulointerstitial network had no significant differences with other nodes (Figure 4A,B). Comparing drug-attacked node in these two different disease networks, drug-attacked nodes in the glomerular network had more important position and attacks to these nodes will more greatly influence linked nodes and whole network robustness, but there was no sizeable difference in the tubulointerstitial network.

### QDZJN Tent to Disturb Glomerular Network of HN Patients Based on Network Robustness

Next, the robustness of whole networks against drug attack was assessed to evaluate QDZJN attack on the disease network of glomeruli and tubules (Figure 4C). Here, two of the most robust measures of network topology, average length of shortest path (APL_net_) and CC_net_, were used. The results showed that overall network structure in the tubulointerstitial network was more robust than that of the random network after drug attack with minimal change in APL_net_ and CC_net_ (Figure 4D,E). However, the network structure of glomerular network was less robust than that of the random network under drug attack with bigger change in APL_net_ and CC_net_ (Figure 4D,E). This finding indicated that drug attack on the disease networks of two renal regions result in a different reaction of genes connection in glomeruli and tubules: the glomerular network was more sensitive, but the tubulointerstitial network remained stable in structure due to the removal of drug targets.

Interestingly, APL_net_ was decreased in the tubulointerstitial network (*p*-value < 0.001 by permutation test) but increased in the glomerular network (*p*-value < 0.001 by permutation test) after drug attack. The different change in APL_net_ reflected the underlying difference in network organization for the two renal regions; drug attack on the glomerulus system was characterized by greater rich-club organization, and increasing dependence on hub nodes, resulting in greater fragility under drug attack. In contrast, the tubulointerstitial network was less affected due to its redundant structure. Moreover, CC_net_ was only decreased in the glomerular network (*p*-value < 0.001 by permutation test), but not in the tubulointerstitial network (*p*-value > 0.05 by permutation test). The different change in CC_net_ implies that the glomerular system is more sensitive to QDZJN attack. Overall structure changes of glomerular and tubulointerstitial networks after drug attack show that QDZJN specifically targeted glomeruli, as shown by the increased APL_net_ and decreased CC_net_ after removal of drug targets.

### Mechanism of Drug Protection Against Glomerular Damage Induced by Hypertension

**FIGURE 5:**
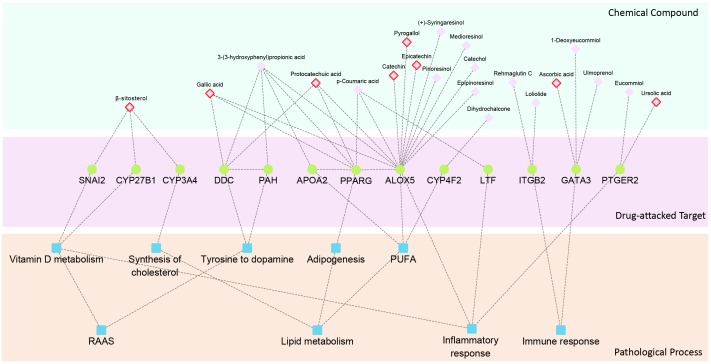
Hierarchy network of chemical compound, drug-attacked nodes in the glomerular network and HN related pathological processes. Compounds with red edge represent drug with anti-hypertensive activity.

To illustrate the mechanism of QDZJN against glomerular damage induced by hypertension, a hierarchical network of chemical compounds common targets of QDZJN and DEGs from glomeruli, and pathological processes related to HN was constructed, including drug-target interactions and relationships between targets and HN pathological processes (Figure 5). This network shows that 13 drug-attacked nodes in the glomerular network were related to hypertensive pathological processes and 21 chemical compounds of QDZJN interacted with these drug-attacked nodes. These 13 drug-attacked nodes belonged to four key pathways of HN, e.g., RAAS, lipid metabolism, immune response, and inflammatory response. PAH interacts with DCC to regulate dopamine synthesis from tyrosine. Recent studies show that the intrarenal dopaminergic system indirectly inhibits renal renin expression (Zhang et al., 2005). Specifically, CYP3A4, CYP27B1, and SNAI2 play important roles in vitamin D metabolism, and evidence has been collected to indicate that vitamin D may interact with renin to represses the RAAS system and reduce the loss of glomerular filtration rate (Santoro et al., 2015). Some drug-attacked nodes maintain lipid metabolic homeostasis (specifically, metabolism of cholesterol and PUFAs, adipogenesis). As a synthesis enzyme of cholesterol, CYP3A4 closely connects to high blood pressure. The SNP genotypes of APOA2, ALOX5, and CYP4F2 were associated with the content of PUFAs (Tagetti et al., 2015; Ballester et al., 2016) which is associated with significant improvement in vascular function and lower blood pressure (Colussi et al., 2016). Epidemiological studies show that circulating PUFAs contribute to preserving renal function (Syren et al., 2017). PPARG influences adipogenesis in glomeruli and knock down of PPARG can lead to glomerular hypertrophy (Toffoli et al., 2017). GATA3, and ITGB2 participated in different immune responses, glomerular immunoglobin A deposition (Yamanaka et al., 2016) and leukocyte recruitment to the inflamed glomerulus (Kuligowski et al., 2006), respectively. In glomerular injury, upregulation of ALOX5 promotes inflammation (Hao and Breyer, 2007), but LTF (Drago-Serrano et al., 2017) and PTGER2 suppress it. As the prostaglandin E2 receptor, activation of PTGER2 accentuates chronic inflammation and protects against angiotensin II-induced hypertension via inhibition of oxidative stress (Jia et al., 2008; Jiang and Dingledine, 2013). Vitamin D also preserves kidney function via attenuating the inflammatory response during lipopolysaccharide-induced acute kidney injury (Xu et al., 2015). Among these drug-attacked nodes, LTF, PPARG, and APOA2 have been proven to be targets for hypertension treatment (Ruiz-Giménez et al., 2012; Ballester et al., 2016; Toffoli et al., 2017). Eight out of 21 possible active compounds have been already found to have anti-hypertension or glomerular protection activity, including ursolic acid (Somova et al., 2003; Zhou et al., 2010), β-sitosterol (Olaiya et al., 2014), ascorbic acid (Duffy et al., 1999), gallic acid (Jin et al., 2017), protocatechuic acid (Safaeian et al., 2016), pyrogallol (Lai and Spector, 1978), epicatechin (Galleano et al., 2013), and catechin (Rhee et al., 2002; Bhardwaj and Khanna, 2013).

## Discussion

As the leading cause of cardiovascular mortality, hypertension also leads to damage of various organs, especially renal damage. As a multicomponent drug, QDZJN was widely used to control blood pressure and protect renal function. In our study, QDZJN was found to specifically target glomerular damage of HN patients.

Hypertensive nephropathy is a medical condition in which chronic high blood pressure causes kidney damage, including vascular, glomerular, and tubulointerstitial lesions. Glomeruli and tubules play different roles in HN. Thus, tubulointerstitial changes are regarded as a major determinant in the progression of renal damage and may be the important initial sites of injury. Then, an increase in capillary pressure causes segmental necrosis and glomerulosclerosis. Finally, glomerulosclerosis and preglomerular vascular structural alterations can cause a further reduction in renal blood flow. Based on transcriptome data from Berthier’s study, DEGs in glomeruli specifically participate in pathways related to blood pressure control and cholesterol homeostasis. Glomeruli and tubules also share some pathways, including oxidative stress, angiogenesis, inflammatory response and immune response.

**FIGURE 6:**
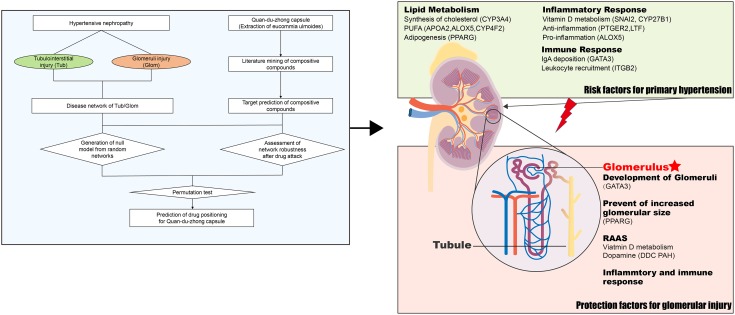
QDZJN against HN via renal protection and reduction of hypertension risk. Left part is the schematic diagram of tissue location of drug based on network robustness. Possible mechanism of QDZJN protect against glomerular injury of HN was summarized in right part.

Because of the anti-oxidant, anti-hypertensive, and anti-inflammatory activities of QDZJN, it has the potential to treat HN. Moreover, potential targets of QDZJN were specifically expressed in renal tissue, which provides more evidence to support QDZJN positioning to HN. If QDZJN has the potential to prevent renal damage, question include how and on which part QDZJN will intervene. To discovery the precise target sub-organ position of QDZJN, the robustness of disease networks from glomeruli and tubules was evaluated under QDZJN attack (Figure 6). Drug-attacked nodes in the glomerular network had more important positions in disease network with larger CC_node_ and smaller shortest path length, which implies that attack to these nodes will more strongly influence network robustness. Overall structural change in the glomerular and tubulointerstitial networks after QDZJN attack show that QDZJN specifically target glomeruli, as indicated by the increased shortest path length and decreased CC_net_ of the whole network after removal of drug targets.

A hierarchical network of 21 chemical compounds of QDZJN, 13 drug-attacked nodes in the glomerular network, and pathological processes related to HN was constructed to clarify the relationship between drug and disease. And all these 21 possible active compounds may target on 13 genes specifically expressed in glomeruli which were related to RAAS, lipid metabolism, immune response, and inflammatory response. These biological processes can be divided into two categories, primary hypertension factors and kidney protection factors (Figure 6). Specifically, lipid metabolism is a critical pathway in primary hypertension and plays important roles in renal damage. CYP3A4, APOA2, PPARG, ALOX, and CYP4F2, proteins related to lipid homeostasis affect the occurrence of hypertension, and PTGER2, ALOX5, LTF, ITGB, and GATA3 relate to the inflammatory and immune response in glomeruli. For kidney protection, GATA3 and PPARG both have protective effects on glomeruli. GATA3 was specifically expressed in glomeruli and is related to renal aplasia (Moriguchi et al., 2016, 3). PPARG influences adipogenesis in glomeruli, and knock down of PPARG can lead to glomerular hypertrophy (Toffoli et al., 2017). SNAI2, CYP27B1, DDC, and PAH inhibits the RAAS system to reserve glomerular function by regulating concertation of vitamin D and dopamine. In summary, QDZJN can preserve renal function and reduce hypertensive risk factor by anti-inflammation, antioxidation and regulation of metabolic homeostasis.

Similar to QDZJN, most multicomponent drugs have various medicinal properties, making it a challenge to maximize the efficacy of drugs and to find optimum indications. Based on the “multi-component and multi-target” principle, multiple attacks on network can simulate multicomponent drug effects to disease network. Evaluation of network robustness can assess the strength of drug attacks on network. In previous studies, network robustness has been used in drug discovery, and cancer biology. In this study, we innovatively proposed a strategy to precisely position the clinical application of drug based on network robustness which can be applied to the precise clinical positioning of other multi-target drugs. This strategy suggests a new approach for multicomponent drug discovery. For multi-target drugs, including monomers with many targets, and multicomponent drugs, approaches based on network robustness can be applied to many research topics, such as, the best indication for a drug and, upon which part of a complex disease does the drug work. In our study, QDZJN was repositioned to HN, especially glomeruli, which is also a new finding about QDZJN and needs further experimental verification. Prediction of precise position for drug is very helpful for drug clinical applications, QDZJN may be used for patients with glomerular injury for better pharmaceutical effects.

## Author Contributions

FG, HX, and HY conceived the study and wrote the manuscript. FG and WZ performed the data analysis. JS collected the component of QDZJN. All authors reviewed and approved the final manuscript.

## Conflict of Interest Statement

The authors declare that the research was conducted in the absence of any commercial or financial relationships that could be construed as a potential conflict of interest.

## Footnotes

1 http://www.chemspider.com/

## Supplementary Material

TABLE S1 Component compounds of QDZJN by literature mining.

## Abbreviations

APL: average length of shortest path
CC_net_: clustering coefficient
CC_node_: closeness centrality
CFDA: China’s Food and Drug Administration
DEGs: differentially expressed genes
EU: *Eucommia ulmoides*
HN: hypertensive nephropathy
PUFAs: polyunsaturated fatty acids
QDZJN: Quan-du-zhong capsule
RAAS: renin-angiotensin-aldosterone system
ROS: reactive oxygen species
